# Supplementary material for: HIVprotI: an integrated web based platform for prediction and design of HIV proteins inhibitors
Source: J Cheminform. 2018 Mar 9;10:12. doi: 10.1186/s13321-018-0266-y (PMC5845081; doi:10.1186/s13321-018-0266-y)
Supplement: Supplementary file 2 — Additional file 2. Source code of HIVProtI web server. [file 13321_2018_266_MOESM2_ESM.zip › HIVprotI_Source-code/hivprotI/weka-3-6-6/doc/weka/datagenerators/package-summary.html]

weka.datagenerators


---


|  |  |  |
| --- | --- | --- |
| **Overview** \| **Package** \| Class \| **Tree** \| **Deprecated** \| **Index** \| **Help** \| **Weka's home** | |  |
| **PREV PACKAGE**   **NEXT PACKAGE** | **FRAMES**    **NO FRAMES**     **All Classes** |  |


---

## Package weka.datagenerators

| **Class Summary** | |
| --- | --- |
| **ClassificationGenerator** | Abstract class for data generators for classifiers. |
| **ClusterDefinition** | Ancestor to all ClusterDefinitions, i.e., subclasses that handle their own parameters that the cluster generator only passes on. |
| **ClusterGenerator** | Abstract class for cluster data generators. |
| **DataGenerator** | Abstract superclass for data generators that generate data for classifiers and clusterers. |
| **RegressionGenerator** | Abstract class for data generators for regression classifiers. |
| **Test** | Class to represent a test. |

---


|  |  |  |
| --- | --- | --- |
| **Overview** \| **Package** \| Class \| **Tree** \| **Deprecated** \| **Index** \| **Help** \| **Weka's home** | |  |
| **PREV PACKAGE**   **NEXT PACKAGE** | **FRAMES**    **NO FRAMES**     **All Classes** |  |


---
